# Simulation-Based Training of the Rapid Evaluation and Management of Acute Stroke (STREAM)—A Prospective Single-Arm Multicenter Trial

**DOI:** 10.3389/fneur.2019.00969

**Published:** 2019-09-11

**Authors:** Ferdinand O. Bohmann, Natalia Kurka, Richard du Mesnil de Rochemont, Katharina Gruber, Joachim Guenther, Peter Rostek, Heike Rai, Philipp Zickler, Michael Ertl, Ansgar Berlis, Sven Poli, Annerose Mengel, Peter Ringleb, Simon Nagel, Johannes Pfaff, Frank A. Wollenweber, Lars Kellert, Moriz Herzberg, Luzie Koehler, Karl Georg Haeusler, Anna Alegiani, Charlotte Schubert, Caspar Brekenfeld, Christopher E. J. Doppler, Oezguer A. Onur, Christoph Kabbasch, Tanja Manser, Waltraud Pfeilschifter, STREAM Trial Investigators, Mohammad Alotaibi

**Affiliations:** University Hospital Frankfurt, Theodor-Stern-Kai 7, 60590 Frankfurt am Main, Germany: Department of Neurology, University Hospital Frankfurt; Department of Neurology, University Hospital Frankfurt; Department of Neurology, University Hospital Frankfurt; Department of Neurology, University Hospital Frankfurt; Department of Neurology, University Hospital Frankfurt; Department of Neurology, University Hospital Frankfurt; Department of Neurology, University Hospital Frankfurt; Department of Neurology, University Hospital Frankfurt; Department of Neurology, University Hospital Frankfurt; Department of Neurology, University Hospital Frankfurt; Institute for Diagnostic and Interventional Neuroradiology, University Hospital Frankfurt; Klinikum Augsburg, Department of Neurology and Clinical Neurophysiology, Stenglinstr. 2 86156 Augsburg, Germany: Department of Neurology and Clinical Neurophysiology, Klinikum Augsburg; Department of Neurology and Clinical Neurophysiology, Klinikum Augsburg; University Hospital Tübingen, Hoppe-Seyler-Str. 3, 72076 Tübingen, Germany: Department of Neurology with Focus on Neurovascular Diseases and Neurooncology, and Hertie Institute for Clinical Brain Research, University of Tübingen; Department of Neurology with Focus on Neurovascular Diseases and Neurooncology, and Hertie Institute for Clinical Brain Research, University of Tübingen; Department of Neurology with Focus on Neurovascular Diseases and Neurooncology, and Hertie Institute for Clinical Brain Research, University of Tübingen; University Hospital Heidelberg, Im Neuenheimer Feld 400, 69120 Heidelberg, Germany: Department of Neurology, University Hospital Heidelberg; Department of Neurology, University Hospital Heidelberg; Department of Neurology, University Hospital Heidelberg; Department of Neurology, University Hospital Heidelberg; Department of Neurology, University Hospital Heidelberg; Department of Neurology, University Hospital Heidelberg; Department of Neurology, University Hospital Heidelberg; Department of Neurology, University Hospital Heidelberg; Department of Neurology, University Hospital Heidelberg; Department of Neurology, University Hospital Heidelberg; Department of Neurology, University Hospital Heidelberg; Department of Neuroradiology, University Hospital Heidelberg; Ludwig Maximilians-University, Marchioninistrasse 15, 81377 Munich, Germany: Department of Neurology, Ludwig Maximilians-University, Munich; Department of Neurology, Ludwig Maximilians-University, Munich; Department of Neurology, Ludwig Maximilians-University, Munich; Department of Neurology, Ludwig Maximilians-University, Munich; Department of Neurology, Ludwig Maximilians-University, Munich; Department of Anesthesiology, Ludwig Maximilians-University Munich; Department of Neurology, Ludwig Maximilians-University, Munich; Department of Neurology, Ludwig Maximilians-University, Munich; Department of Neurology, Ludwig Maximilians-University, Munich; Department for Diagnostic and Interventional Neuroradiology of the University of Munich (LMU), Campus Grosshadern; Department for Diagnostic and Interventional Neuroradiology of the University of Munich (LMU), Campus Grosshadern; Department for Diagnostic and Interventional Neuroradiology of the University of Munich (LMU), Campus Grosshadern; Institute for Stroke and Dementia Research, Ludwig Maximilians-University Munich; Institute for Stroke and Dementia Research, Ludwig Maximilians-University Munich; Emergency Department, Klinikum Grosshadern, Ludwig Maximilians University, Munich; Charité - University Hospital Berlin, Center for Stroke Research Berlin (CSB), Hindenburgdamm 30, 12203 Berlin: Department of Neurology, Charité - University Hospital Berlin; Center for Stroke Research Berlin (CSB), Charité - University Hospital Berlin; Center for Stroke Research Berlin (CSB), Charité - University Hospital Berlin; Center for Stroke Research Berlin (CSB), Charité - University Hospital Berlin; Center for Stroke Research Berlin (CSB), Charité - University Hospital Berlin; Center for Stroke Research Berlin (CSB), Charité - University Hospital Berlin; Center for Stroke Research Berlin (CSB), Charité - University Hospital Berlin; Center for Stroke Research Berlin (CSB), Charité - University Hospital Berlin; Center for Stroke Research Berlin (CSB), Charité - University Hospital Berlin; University Medical Centre Hamburg Eppendorf, Martinistr. 52, 20246 Hamburg, Germany: Central Emergency Department, University Medical Centre Hamburg Eppendorf, Hamburg; Department of Diagnostic and Interventional Neuroradiology, University Medical Centre Hamburg Eppendorf, Hamburg; Department of Diagnostic and Interventional Neuroradiology, University Medical Centre Hamburg Eppendorf, Hamburg; Department of Diagnostic and Interventional Neuroradiology, University Medical Centre Hamburg Eppendorf, Hamburg; Department of Neurology, University Medical Centre Hamburg Eppendorf, Hamburg; Department of Diagnostic and Interventional Neuroradiology, University Medical Centre Hamburg Eppendorf, Hamburg; Department of Diagnostic and Interventional Neuroradiology, University Medical Centre Hamburg Eppendorf, Hamburg; Department of Neurology, University Medical Centre Hamburg Eppendorf, Hamburg; Department of Diagnostic and Interventional Neuroradiology, University Medical Centre Hamburg Eppendorf, Hamburg; MD, Department of Diagnostic and Interventional Neuroradiology, University Medical Centre Hamburg Eppendorf, Hamburg; Department of Neurology, University Medical Center Hamburg Eppendorf, Hamburg; Department of Diagnostic and Interventional Neuroradiology, University Medical Centre Hamburg Eppendorf, Hamburg; Department of Diagnostic and Interventional Neuroradiology, University Medical Centre Hamburg Eppendorf, Hamburg; Department of Neurology, University Medical Center Hamburg Eppendorf, Hamburg; Department of Diagnostic and Interventional Neuroradiology, University Medical Centre Hamburg Eppendorf, Hamburg; Department of Neurology, University Medical Center Hamburg-Eppendorf, Hamburg, Germany; Department for Neuroradiological Diagnosis and Intervention, University Medical Center Hamburg-Eppendorf; Department of Neurology, University Medical Center Hamburg-Eppendorf, Hamburg, Germany; Department of Diagnostic and Interventional Neuroradiology, University Medical Centre Hamburg Eppendorf, Hamburg; University Hospital Cologne, Kerpener Str. 62, 50937 Cologne, Germany: Department of Neurology, University Hospital Cologne; Department of Neurology, University Hospital Cologne; Department of Neurology, University Hospital Cologne; Department of Neurology, University Hospital Cologne; PhD, Department of Neurology, University Hospital Cologne; Department of Neurology, University Hospital Cologne; Department of Neurology, University Hospital Cologne; Department of Neurology, University Hospital Cologne; Department of Neurology, University Hospital Cologne; Department of Neurology, University Hospital Cologne; Department of Neurology, University Hospital Cologne; Department of Neurology, University Hospital Cologne; Department of Neuroradiology, University Hospital Cologne; Department of Neuroradiology, University Hospital Cologne;; ^1^Department of Neurology, University Hospital Frankfurt, Frankfurt, Germany; ^2^Institute for Diagnostic and Interventional Neuroradiology, University Hospital Frankfurt, Frankfurt, Germany; ^3^Department of Neurology, Klinikum Hanau, Hanau, Germany; ^4^NICU Nursing Staff, University Hospital Frankfurt, Frankfurt, Germany; ^5^Department of Neurology and Clinical Neurophysiology, Universitätsklinikum Augsburg, Augsburg, Germany; ^6^Department for Diagnostic and Interventional Radiology and Neuroradiology, Universitätsklinikum Augsburg, Augsburg, Germany; ^7^Department of Neurology With Focus on Neurovascular Diseases and Neurooncology, University Hospital Tübingen, Tübingen, Germany; ^8^Hertie Institute for Clinical Brain Research, University of Tübingen, Tübingen, Germany; ^9^Department of Neurology, University Hospital Heidelberg, Heidelberg, Germany; ^10^Department of Neuroradiology, University Hospital Heidelberg, Heidelberg, Germany; ^11^Department of Neurology, Ludwig Maximilians-University, Munich, Germany; ^12^Department for Diagnostic and Interventional Neuroradiology, Ludwig Maximilians-University, Munich, Germany; ^13^Department of Neurology, University Hospital Leipzig, Leipzig, Germany; ^14^Centre for Stroke Research Berlin, Charité—Universitätsmedizin Berlin, Berlin, Germany; ^15^Department of Neurology, Universitätsklinikum Würzburg, Würzburg, Germany; ^16^Department of Neurology, University Medical Centre Hamburg Eppendorf, Hamburg, Germany; ^17^Department of Diagnostic and Interventional Neuroradiology, University Medical Centre Hamburg Eppendorf, Hamburg, Germany; ^18^Department of Neurology, University Hospital Cologne, Cologne, Germany; ^19^Department of Neuroradiology, University Hospital Cologne, Cologne, Germany; ^20^School of Applied Psychology, FHNW University of Applied Sciences and Arts Northwestern Switzerland, Olten, Switzerland

**Keywords:** CRM, thrombolysis (tPA), stroke, emergency care, simulation training

## Abstract

**Introduction:** Acute stroke care delivered by interdisciplinary teams is time-sensitive. Simulation-based team training is a promising tool to improve team performance in medical operations. It has the potential to improve process times, team communication, patient safety, and staff satisfaction. We aim to assess whether a multi-level approach consisting of a stringent workflow revision based on peer-to-peer review and 2–3 one-day *in situ* simulation trainings can improve acute stroke care processing times in high volume neurocenters within a 6 months period.

**Methods and Analysis:** The trial is being carried out in a pre-test-post-test design at 7 tertiary care university hospital neurocenters in Germany. The intervention is directed at the interdisciplinary multiprofessional stroke teams. Before and after the intervention, process times of all direct-to-center stroke patients receiving IV thrombolysis (IVT) and/or endovascular therapy (EVT) will be recorded. The primary outcome measure will be the “door-to-needle” time of all consecutive stroke patients directly admitted to the neurocenters who receive IVT. Secondary outcome measures will be intervention-related process times of the fraction of patients undergoing EVT and effects on team communication, perceived patient safety, and staff satisfaction via a staff questionnaire.

**Interventions:** We are applying a multi-level intervention in cooperation with three “STREAM multipliers” from each center. First step is a central meeting of the multipliers at the sponsor's institution with the purposes of algorithm review in a peer-to-peer process that is recorded in a protocol and an introduction to the principles of simulation training and debriefing as well as crew resource management and team communication. Thereafter, the multipliers cooperate with the stroke team trainers from the sponsor's institution to plan and execute 2–3 one-day simulation courses *in situ* in the emergency department and CT room of the trial centers whereupon they receive teaching materials to perpetuate the trainings.

**Clinical Trial Registration:** STREAM is a registered trial at https://clinicaltrials.gov/ct2/show/NCT03228251.

## Introduction

The benefits of acute stroke therapy critically depend on the time to recanalization of the occluded brain vessel. This has been consistently shown for intravenous thrombolysis (IVT) (1) and endovascular thrombectomy (EVT) (2), nowadays applied to 5–25 or 5–10% of all patients suffering from an ischemic stroke, respectively. Each minute lost reduces therapeutic efficacy and increases the risk of complications (3, 4). When implementing these therapies into daily clinical practice, stroke teams have to strive for optimal process times in order to translate the benefits shown in clinical trials into good outcomes for the patients. Since the widespread introduction of EVT, the chain of acute stroke treatment has become more complex. The patient is cared for by an interdisciplinary team (5) and has to undergo several handovers with possible interface problems.

As treatment delays, errors or misjudgements may put the patient at risk, the initial assessment, diagnostic procedures and therapeutic decisions have to be as fast as possible without losing accuracy. The decision to start IVT and/or EVT should be completed within <30 min, which requires a well-functioning workflow including initial assessment, blood sampling, the organization of further processes, transport, and radiological diagnostics. Recent reports show that excellent process times can be reached at high volume centers but not as reliably at the majority of smaller stroke units worldwide (6, 7). So far, reports on workflow improvement in the setting of acute stroke care have focussed on streamlining of the process but how the teams that work along these processes are actually trained has not been detailed (8, 9). It is a well-recognized phenomenon that time pressure increases the risk of medical errors and puts strain on team communication. Complications and adverse events during medical treatment are a frequent problem (10). In 2010, up to every fourth patient treated in a hospital may have suffered from avoidable medical complications (11, 12). Medical complications caused by human failure are estimated to represent 3–4% of the health expenditure. Approximately 70% of these cases are attributed to human factors, such as miscommunication, forgotten or falsely executed orders, mix-ups, or wrong decisions (13).

One means to counteract the deleterious effect of time pressure on team communication is crew or crisis resource management (CRM), which is a work and training philosophy combining different strategies for teams focusing on non-technical skills in high-reliability environments where human error can have a devastating effect (14). CRM concepts, which were originally designed for aviation, have been modified and adapted to a wide range of high-reliability environments where people have to make time-critical wide-ranging decisions, such as in emergency medicine. Previously, the trial sponsor (University Hospital Frankfurt, Germany) designed a stroke team training for the interdisciplinary multiprofessional team of its neurocenter using an *in situ* simulation along the entire chain of acute stroke care from the paramedics' handover to the beginning of the neurovascular intervention. We introduced the principles of CRM into our stroke care protocol with a focus on communication skills, a defined team, a binding algorithm involving the specific division of tasks and predefined “check points” for a brief team time-out (15). In order to acquaint the staff with the algorithm, we implemented monthly simulation training with a high-fidelity manikin. This intervention was highly efficient (16). We transferred a trimmed-down version of the intervention—relying on a peer-to-peer review of the respective acute stroke algorithms and a one-time simulation training—to our regional stroke network consisting of stroke units of different certification levels where it significantly reduced the network-wide median door-to-needle time in a pre-test-post-test analysis (17).

The STREAM trial is directed at high level stroke centers with 24/7 EVT capacity in a controlled prospective pre-test-post-test design. It will assess the immediate effect of a composite intervention that includes a peer-to-peer review of each center's acute stroke algorithm, which defines the team's composition and details the specific tasks of each team member. This is in combination with a short course of repetitive stroke team simulation training. We hypothesize that the implementation of a stroke team algorithm (defined team with precisely defined tasks), applying the principles of crew resource management (CRM) and stroke team simulation training with a focus on efficient teamwork and communication will improve the processing time, patient safety, and staff satisfaction.

## Methods and Analysis

### Design

**Figure 1:**
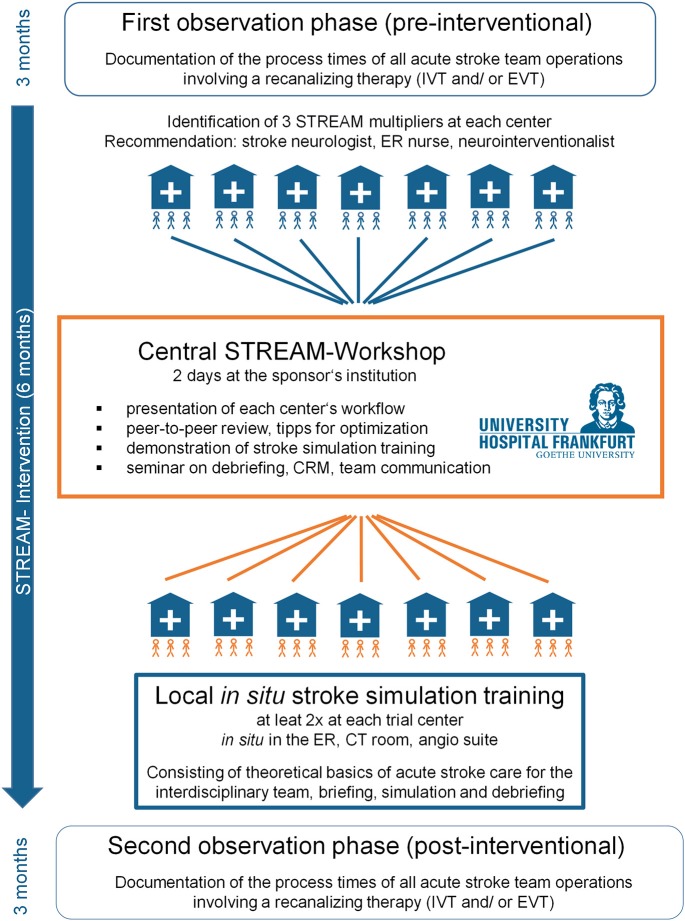
Timeline of the STREAM trial.

STREAM is a prospective single-arm multicenter trial with a pre-test-post-test design (Figure 1) and central data monitoring, employing individual patients' case report forms (Supplemental Data 1) to record the baseline variables, processing time, time-consuming items in the acute stroke workflow, and the team's composition. Additionally, staff questionnaires will be used to capture satisfaction regarding interdisciplinary teamwork, safety climate, stress recognition and error handling as well as general job satisfaction and identification within the workplace.

### Interventional Methods

During a 3-months pre-test period, the participating seven stroke centers (tertiary care university hospitals with 24/7 thrombectomy, well-comparable to other tertiary stroke care centers) will record the data of all consecutive stroke patients directly admitted to the emergency departments. The participating centers are University Hospital Augsburg, University Hospital Tübingen, University Hospital Heidelberg, Ludwig Maximilians-University Munich, Center for Stroke Research Berlin Charité, University Medical Center Hamburg Eppendorf, University Hospital Cologne. University Hospital Frankfurt (Goethe University) is the sponsor of the trial and is not participating in the trial. All consecutive patients undergoing acute recanalization therapy (IVT and/or EVT) will be enrolled. Pre-test staff satisfaction and patient safety will be measured using a 67-item questionnaire, based on the Safety Attitudes Questionnaire (SAQ), a validated instrument used to measure the patient safety climate in clinical areas (18, 19). After the pre-test period (observation phase 1), the data acquisition will be paused for 6 months during which the intervention will take place.

We will perform a multi-level intervention in close cooperation with three “STREAM multipliers” per center. We recommended the centers to nominate one stroke neurologist, one neurointerventionalist and one nurse of the emergency department but left the final decision about the composition of the multiplier team to each center. The first step of the intervention will be a central meeting at the sponsor's institution (to accommodate shift plans etc., we will conduct two meetings with identical programmes and final written distribution of all protocols). The purpose of this first meeting will be a critical revision of each center's acute stroke algorithm in a peer-to-peer process that is captured in a protocol and provided to each center as well as a familiarization of the multiplier team with stroke simulation. To this end, there will be a demonstration of stroke team simulation and a seminar on basic principles of simulation training and debriefing as well as CRM and team communication. The protocol on each center's algorithm will encourage the implementation of an interdisciplinary stroke team with a binding and precise definition and assignment of tasks. Thereafter, the multipliers will cooperate with the sponsor's stroke team trainers to organize 2–3 simulation-based team trainings with a high-fidelity manikin *in situ* in each center's emergency department and CT room. Two of the training sessions shall be conducted by the stroke team trainers from the sponsor while the third training session will be encouraged to be led by one of the multipliers. This is with the aim of establishing the concept of stroke team training and simulation on a regular basis. The centers will be provided teaching materials for use at their institution and their regional stroke networks. The intervention shall be completed within 6 months.

During the following 3-month post-test period (observation phase 2), the participating seven centers will record the process times of all consecutive direct-to-center patients receiving recanalization therapy (IVT and/or EVT). Post-interventional staff satisfaction and perceived patient safety will be measured with a 67-item questionnaire.

### Selection of Centers and Subjects

Centers were selected after a site selection screening taking into account the number of recanalization therapies per year, median process times and the readiness to participate with dedication in the intervention. Randomization into an instant and a delayed intervention cohort would be desirable. However, due to the limited number of stroke centers (*n* = 7) and their heterogeneity, this approach is not feasible. Therefore, all seven centers will undergo the intervention and observation phases simultaneously and a comparison will be made between the pre-test-post-test results of all seven centers grouped together.

All patients over the age of 18 years who are admitted directly to the participating centers and receive IVT (primary endpoint: door-to-needle time) in the context of an acute stroke are eligible irrespective of whether they receive additional EVT or not. If EVT is performed, then the EVT-specific processing time will also be acquired (secondary endpoints). Written informed consent will be obtained from all patients or their legal representatives before the transfer of data to the sponsor. Patients who had been secondarily transferred to the stroke centers for EVT (“drip-and-ship”) and patients suffering in-hospital stroke will be excluded from the trial.

Observation phase 1: All direct-to-center stroke patients receiving thrombolysis and/or thrombectomy in the 3 months before the intervention phase.

Observation phase 2: All direct-to-center stroke patients receiving thrombolysis and/or thrombectomy in the 3 months after the intervention phase.

### Data Analysis

The predefined primary endpoint is the median time from the arrival of the stroke patients until the administration of the alteplase bolus (“door-to-needle” time) of all consecutive direct-to-center stroke patients in observation periods 1 and 2 is the primary endpoint.

The predefined secondary endpoints are median EVT process time (“door-to-angio” comprising the critical handover to the anesthesia or neurocritical care team and “door-to-groin” comprising the entire process up to the endovascular procedure). Besides that, secondary haemorrhagic transformation (symptomatic or asymptomatic) will be recorded as a safety endpoint. The team composition, as well as any time-consuming processes, will also be recorded as crucial variables targeted by our intervention. Via the questionnaires, we aim to assess interdisciplinary teamwork, the safety climate, stress recognition and error handling as well as staff satisfaction and identification with the workplace during both observation periods.

All data specified in the trial protocol will be documented in standardized Case Report Forms (CRFs, Supplemental Data 1) by the participating stroke centers. Data validation, including the control of completeness, consistency and plausibility of the data documented in the CRF, is the responsibility of the local principal investigator (PI) and the documenting investigators. HR, who is a clinical trials coordinator at the sponsor's institution, will carry out source data verification of 15% of recruited patients.

A pilot trial on the efficacy of a fixed stroke team algorithm and regular simulation-based stroke team training in our regional stroke network consisting of 7 stroke units showed a shortening of the network-wide median “door-to-needle” time by 12 min (43, IQR 30–60 to 31, IQR 24–42 min). A priori sample size calculation based on this data yielded a minimal number of 110 patients in each group to prove the comparative efficacy of the composite intervention on the primary endpoint with a statistical power of 0.8 and a type 1 error probability of <0.05. The seven tertiary care stroke centers perform 80–250 thrombolyses per year and should jointly be able to recruit 200 patients in each intervention phase considering the potential dropout rate for missing reports or informed consent in 1/3 of the patients. The centers will be explicitly motivated to record all consecutive patients and they will be obliged to prove that adequate efforts have been made via a monitored screening log.

We will present the median and 25–75 percent interquartile ranges (IQR) of the process times and test differences between the intervention phases for statistical significance via the Wilcoxon-Mann-Whitney Test. The answers provided within the questionnaire capturing satisfaction with the interdisciplinary teamwork, safety climate, stress recognition and error handling, as well as staff satisfaction and identification with the workplace, will be recorded using an ordinal 5-point Likert scale.

## Discussion

The STREAM trial will evaluate the effect of a five-step intervention for acute stroke therapy on process times, staff satisfaction and perceived patient safety. The intervention aims at optimizing and standardizing stroke treatment by applying aspects of crew resource management (CRM) and simulation training adapted to acute stroke care. It may provide evidence of efficacy for this composite intervention that could pave the way for the implementation of structured team trainings for acute stroke care, as they are already practiced in other medical disciplines.

In high-reliability environments where decisions are time-critical, structured algorithms based on the principles of crew-resource management help to improve patient safety. Once developed, these SOPs have to be introduced to the entire staff. We hypothesize that simulation training will facilitate the transfer of the new algorithms by allowing experiential learning and self-reflection. To evaluate the safety of our intervention, which aims at accelarating IVT, we will capture intra- and extracranial haemorrhagic complications and will compare the frequency between the pre- and post-interventional phases.

Structured team training based on crew resource management (CRM) has been successfully implemented into emergency medicine, intensive care and surgery. In these contexts, training has led to a significant fall in the mortality rate (up to 18%) (20, 21). To our knowledge, there have been only a few reports on the effect of simulation training on process times in acute stroke care. Existing studies rely on one-time interventions at a simulation facility (22), trained hospitals with little prior experience (23) and/or used retrospective case analyses as a comparator (23, 24). We have previously applied the stroke team approach to the sponsor's stroke admissions team (16) and consequently to our regional stroke network (17), which at the time consisted of seven stroke units. In that study, we found that the intervention (algorithm design, introducing CRM, and simulation training) was effective in reducing the network wide door-to-needle time from a median of 43 min (IQR 30–60 min) to 31 min (IQR 24–42 min) (16) and door-to-groin-time from 59 min (IQR 35–102 min) to 43 min (IQR 28–81 min) (25). However, these studies were limited to a regional network consisting of heterogeneous stroke units of different sizes with and without the capacity for EVT. The multicentric study in the stroke network relied on data entry by the local investigators without central source data verification. By testing the stroke team concept in seven high volume academic tertiary care stroke centers with the 24/7 capacity for thrombectomy in a multicentric pre-test-post-test study, we aim to control some of these limitations and measure the effectiveness of the intervention more reliably. A cluster randomization by center would have been desirable, but the heterogeneity in between centers (e.g., hospital infrastructure, rural vs. urban catchment area, case load, and percentage of drip-and-ship patients) does not permit this. Therefore, we opted for a prospective pre-test-post-test design with central source data monitoring.

Another question is the scalability of our composite intervention. In our regional stroke network, stroke team training could be introduced on a regular basis following the above-named effectiveness study (16). So far, trainings are carried out as part of the cooperation between stroke team trainers from the university hospital with the senior staff of local stroke units. Obstacles to a decentralized implementation of an independent regular training at each site include time and access to a qualification programme for stroke team trainers and to stroke specific teaching materials such as simulation equipment, slide-kits and scripted scenarios. If the STREAM trial demonstrates effectiveness of the intervention, it may further promote stroke team simulation as a versatile tool for the education of inter-professional teams. Since all hospitals participating in this trial are part of and often coordinate regional stroke networks, the intervention could be secondarily propagated to these stroke networks. From the feedback of the participating centers, we hope to get an insight into the possible difficulties faced in the implementation processes.

We acknowledge that there are a few limitations that we could not circumvent when designing this trial: randomization is not possible due to the limited number of centers. Since we targeted high volume centers with a median door-to-needle time of 30 min and below, it will be challenging to show an improvement in the primary endpoint. The success of the intervention will crucially depend on the cooperation of all involved medical disciplines and professionals—a factor that is not easy to foresee in the planning phase of a trial. It will probably not be possible to train all staff members involved in acute stroke therapy during the three *in situ* simulations at each center, so we will have to rely on a snowball effect. Notably, including stroke patients who are able to provide written informed consent or stroke patients with a legal representative implies a selection bias. Finally, it will not be possible to discern the impact of the different levels of our intervention.

Nevertheless, we think that the results of this pragmatic and rigorously controlled trial of workflow-improvement and *in situ* simulation could have an impact on the standards of acute stroke care and the training of junior staff in this field.

## Ethics Statement

STREAM is a registered trial at study PI (WP) and all local PIs are https://clinicaltrials.gov/ct2/show/NCT03228251. The trial was approved by the institutional ethics review board of the University Hospital Frankfurt with secondary approval by the local IRBs of all participating centers. The trained according to the needed certification in the context of practice. They are able conduct clinical trials according to Good Clinical Practice (GCP) and the Declaration of Helsinki. The study was funded by Stryker Neurovascular. The funding source was not involved in study design, monitoring, data collection, statistical analyses, interpretation of results, or manuscript writing. The trial results will be disseminated at national and international conferences (Neurowoche, European Stroke Conference) and we aim at publication in an international peer-reviewed journal. De-identified patient-level data can be made available upon well-founded request to the corresponding author after clearance with the ethics review board of the University Hospital Frankfurt.

## Author Contributions

FB, NK, PRo, HR, and WP: conception of the trial. FB, NK, and WP: drafting of the manuscript and design of the trial intervention. FB, NK, HR, KG, JG, TM, and WP: design of case report form and questionnaire. KG, HR, and FB: central management of process time data. JG, HR, and FB: central management of staff questionnaire data. RM, PZ, ME, AB, SP, AM, PRi, SN, JP, FW, LKe, MH, LKo, KH, AA, CS, CB, CD, OO, and CK: local data acquisition and analysis at the trial centers. All authors critically revised the manuscript for important intellectual content, provided approval for publication of the content, and agreed to be accountable for all aspects of the work in ensuring that questions related to the accuracy or integrity of any part of the work are appropriately investigated and resolved.

### Conflict of Interest Statement

The authors declare that the research was conducted in the absence of any commercial or financial relationships that could be construed as a potential conflict of interest.

## Supplementary Material

Supplemental Data 1 Case report form of the STREAM trial.
