# Supplementary material for: Simulation-Based Training of the Rapid Evaluation and Management of Acute Stroke (STREAM)—A Prospective Single-Arm Multicenter Trial
Source: Front Neurol. 2019 Sep 11;10:969. doi: 10.3389/fneur.2019.00969 (PMC6749045; doi:10.3389/fneur.2019.00969)
Supplement: Supplemental Data 1 — Case report form of the STREAM trial. [file Table_1.DOCX]

**Pat. ID:** I0I0I1I - I_I_I_I **Year of Birth** I_I_I_I_I

**Index Event:**

| Syptom onset known? | □ Yes : I_I_I I_I_I I_I_I_I_I I_I_I:I_I_I  day month year hh:min |
| --- | --- |
|  | □ No, time of recognition:  I_I_I I_I_I I_I_I_I_I I_I_I:I_I_I  day month year hh:min |
| Hospital admission | I_I_I I_I_I I_I_I_I_I I_I_I:I_I_I  day month year hh:min |
| mRS prior to index stroke | □ 0 □ 1 □ 2 □ 3 □ 4 □ 5 |
| NIHSS at admission | I_I_I |

**Questions regarding stroke diagnostic and therapy**

| CCT: □ yes □ no  CT-Angiography: □ yes □ no | MRT: □ yes □ no  MR-Angiography: □ yes □ no |
| --- | --- |
| Large Vessel Occlusion:  □ no □ yes, if yes -> | □ Carotid-T □ M1 □ M2 □ A1  □ Basilar artery □ Other: ______________ |
| Coagulation-POCT: □ yes □ no |  |
| Thrombolysis: □ yes □ no  if yes, start:  I_I_I I_I_I I_I_I_I_I I_I_I:I_I_I  day month year hh:min | Thrombectomy: □ yes □ no  In-house: □ yes □ no  Arrival time angio suite:  I_I I_I_I I_I_I_I_I I_I_I:I_I_I  day month year hh:min  Time of groin puncture  I_I I_I_I I_I_I_I_I I_I_I:I_I_I  Day month year hh:min  Transfer for thrombectomy: □ yes □ no |

**Pat. ID:** I0I0I1I - I_I_I_I

**Questions regarding STROKE TEAM-Workflow**

| Direct patient transfer from EMS stretcher to CT table: □ yes □ no  Paramedics involved in in-house algorithm: □ yes □ no  Neuroradiologist present during imaging: □ yes □ no  tPA bolus given directly in the CT/MRI : □ yes □ no  Coagulation results awaited before start of thrombolysis: □ yes □ no  Acute medication necessary (e.g. blood pressure treatment) □ yes □ no  Patient needs special care (e.g. vomiting, cardiorespiratory problems) □ yes □ no | |
| --- | --- |
| Number of physicians Neurology I_I_I  Number of physicians (Neuro-)radiology: I_I_I  Number physicians Anaesthesiology: I_I_I  Other _____________ I_I_I  (please specify) | Number of physicians Emergency Unit: I_I_I  Number of Nursing Staff: I_I_I  Number of Paramedics: I_I_I  At least one team member participated before in stroke team training: □ yes □ no |
| Nursing responsibility: □ Emergency Nursing Staff  □ Neurology Nursing Staff  □ “Thrombolysis Nurse” | Medical Responsibility:□ Specialist  □ Medical Specialist in Training  □ Neurology  □ Internal Medicine  □ Anaesthesiology  □ Other: _______________  (please specify) |
| Intracerebral bleeding in follow-up imaging:  □ yes □ no  if yes: clinical symptoms □ yes □ no | Extracerebral bleeding □ yes □ no |

Please send this form to: University Hospital Frankfurt, Department of Neurology Fax: 069/6301 5628 / email: [heike.braun@kgu.de](mailto:heike.braun@kgu.de)
